# Mass spectrometry-based metabolite profiling reveals functional seasonal shifts in the metabolome of *Zygophyllum dumosum Boiss* and its relation to environmental conditions

**DOI:** 10.1007/s00425-023-04168-2

**Published:** 2023-06-03

**Authors:** Noga Sikron-Persi, Gila Granot, Albert Batushansky, David Toubiana, Gideon Grafi, Aaron Fait

**Affiliations:** 1 French Associates Institute for Agriculture and Biotechnology of Drylands, Jacob Blaustein Institutes for Desert Research, Ben-Gurion University of the Negev, Sde Boker Campus, 84990 Midreshet Ben-Gurion, Israel; 2 Ilse Katz Institute for Nanoscale Science and Technology, Ben-Gurion University of the Negev, 84105 Beer Sheva, Israel

**Keywords:** Metabolomics, Arid environment, GC–MS, UPLC–MS-QTOF, Seasonal rhythm

## Abstract

**Main conclusion:**

A multi-year study of perennial *Z. dumosum* shows a consistent seasonal pattern in the changes of petiole metabolism, involving mainly organic acids, polyols, phenylpropanoids, sulfate conjugates, and piperazines.

**Abstract:**

GC–MS and UPLC–QTOF-MS-based metabolite profiling was performed on the petioles of the perennial desert shrub *Zygophyllum dumosum Boiss* (Zygophyllaceae). The petioles, which are physiologically functional throughout the year and, thus, exposed to seasonal rhythms, were collected every month for 3 years from their natural ecosystem on a southeast-facing slope. Results showed a clear multi-year pattern following seasonal successions, despite different climate conditions, i.e., rainy and drought years, throughout the research period. The metabolic pattern of change encompassed an increase in the central metabolites, including most polyols, e.g., stress-related D-pinitol, organic and sugar acids, and in the dominant specialized metabolites, which were tentatively identified as sulfate, flavonoid, and piperazine conjugates during the summer–autumn period, while significantly high levels of free amino acids were detected during the winter–spring period. In parallel, the levels of most sugars (including glucose and fructose) increased in the petioles at the flowering stage at the beginning of the spring, while most of the di- and tri-saccharides accumulated at the beginning of seed development (May–June). Analysis of the conserved seasonal metabolite pattern of change shows that metabolic events are mostly related to the stage of plant development and its interaction with the environment and less to environmental conditions per se.

**Supplementary Information:**

The online version contains supplementary material available at 10.1007/s00425-023-04168-2.

## Introduction

*Zygophyllum dumosum Boiss* is one of more than 80 species from the large genus *Zygophyllum* of the family *Zygophyllaceae* (Sheahan and Cutler 1993). The name *Zygophyllum* describes the two leaflets on the petiole. The petiole seems to play a significant role in plant survival (Sayed 1996). *Z. dumosum* is a Saharo-Arabian perennial shrub dominating the rocky hillslopes of the northern and central Negev Desert in Israel, which is characterized by limited and unpredictable rains (< 100 mm of annual precipitation), extreme temperatures, and high radiation.

**Fig. 1:**
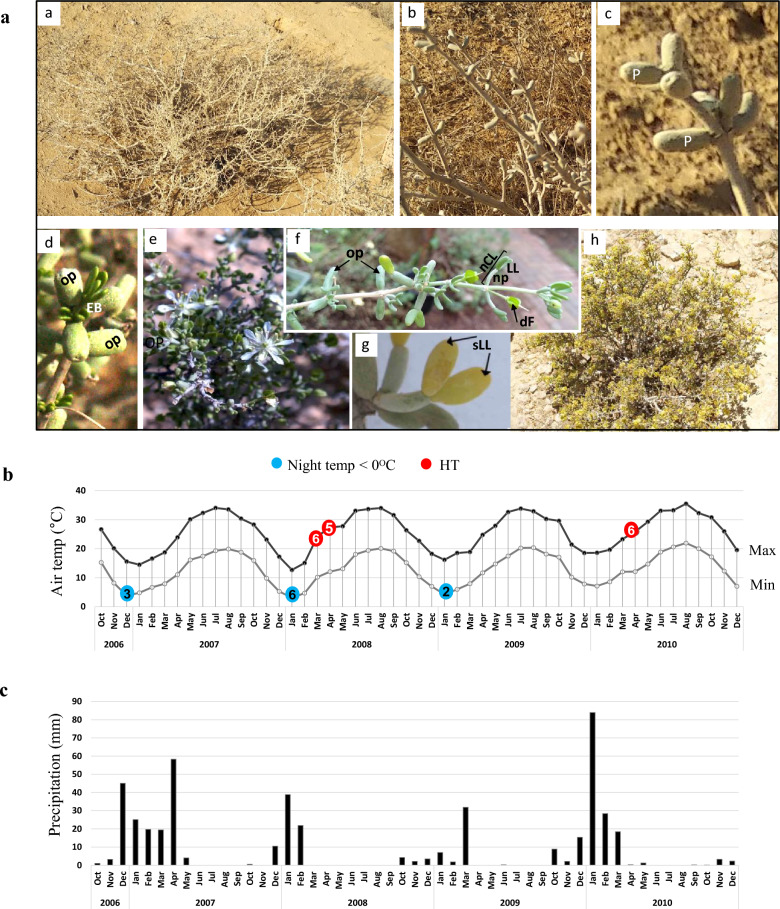
**a** Different growth stages during the year of *Z. dumosum Boiss* (**a–h**). *op* old petiole, *np* new petiole, *EB* early bud, *LL* two leaflets, *nCL* new clause, *dF* dead flower, *sLL* leaflet senesced. **b** Average of monthly, maximal, and minimal air temperatures. Blue circle: number of nights with temperatures below 0 °C, red circle: number of days with HT (high temperature). **c** Amount of monthly precipitation at Sde Boker

The remarkable success of *Z. dumosum* plants is likely due to their wide range of adaptive abilities (Grafi 2019), which include a tight control of germination (Agami 1986), two functional root systems that become active at different depths and times (Rodriguez Zaragoza et al. 2005), and the abscission of leaflets during the dry season (Fig. 1a–c), leading to substantial reduction in the whole-plant transpiration (Terwilliger and Zeroni 1994), while the petioles take over minimal leaf functions. Petioles of *Z. dumosum* can survive for up to two full growing seasons (Schulze et al. 1972). At the molecular level, such halophyte plants are equipped with a powerful antioxidant system, which is responsible for the ability to withstand and quench the toxic reactive oxygen species (ROS) produced under saline conditions (Gharibi et al. 2019; Fabregas and Fernie 2019). Despite these evidence, no studies on the plant’s metabolic adaptation are available, as research has especially aimed at identifying bioactive molecules for potential medical use, such as saponins (Khan et al. 2014). Recently, it was shown that the adaptation of *Z. dumosum* can be mediated via genome modifications (Khadka et al. 2018; Granot et al. 2009). Thus, the transition from the wet to the dry season is associated with a significant reduction in nuclear size, resulting from genome compaction and the gradual decline of histone H3 dimethylated at lysine 4 (H3K4me2), a well-known modification associated with permissive chromatin, which facilitates entry into summer dormancy (Grafi 2019). Notably, species in the *Zygophyllaceae* family, including *Z. dumosum*, *Peganum harmala,* and *Larrea tridentata*, all lack the restrictive histone modification of H3K9me2 (Granot and Grafi 2014), a characteristic feature that might confer Zygophyllaceae species, commonly inhabiting the world’s arid and semi-arid regions, with the capacity for prompt response to changing environments and improved performance in variable, unpredictable habitats.

During the summer, *Z. dumosum*, carrying petioles, enters a dormant-like state, which continues through the autumn (Fig. 1a–c). Resumption of growth at the end of autumn requires input of rain, resulting in the increased size of old petioles and reactivation of photosynthesis, concomitantly with bud emergence and new leaf production (Fig. 1d). Depending on climate conditions, flowering starts during the winter, and blooming starts during the spring, then fruits are produced (Fig. 1e, f), and leaflets are senesced and shed (Fig. 1g, h).

Seasonal changes in climate are known to modulate molecular processes, as well as physicochemical and biological characteristics, reflecting the plant response to the environment (Krasensky and Jonak 2012). However, metabolic adaptation of perennial vegetative tissue to harsh environmental conditions is still poorly understood. This gap in knowledge is becoming critical when considering the impact of climate change on seasonality.

Here, we report the seasonal cycles in the central and the specialized metabolism of *Z. dumosum* petioles and their relation with environmental parameters, revealing the plant’s metabolic adaptation strategy.

## Materials and methods

### Field site and conditions

The study was conducted in the Mount Negev region, in a cleared research area on a southeast-facing rocky slope (30^o^51′N 34^o^46′E; elevation 498 m), close to Ben-Gurion University’s Sde Boker Campus and which is dominated by *Zygophyllum dumosum*. Specific features of the study area have been described elsewhere (Herwitz and Olsvigwhittaker 1989; Terwilliger and Zeroni 1994). The study was conducted in the natural environment, from 2007 to 2010. According to the Israel Meteorological Service, the average annual precipitation in the Sde Boker region is 93 mm and is characterized by high year-to-year variability. Thus, during our study, the precipitation in 2007 and 2010 was 63% and 42% above the average, respectively (Fig. 1c). In contrast, the 2009 precipitation was only 53% of the average (Fig. 1c). Average, monthly maximal, and monthly minimal air temperatures were similar between the years (Fig. 1b) and Relative humidity ranged from 50 to 80% (data in the Supplementary Fig. s2). Global radiation and wind speed were similar between the years (data not shown).

### Plant material extraction of polar metabolites and derivatization for GC–MS and UPLC–QTOF-MS analysis of central and specialized metabolites

Petioles of *Z. dumosum* were collected every month during 4 consecutive years (2007–2010), except for July of 2007, and April, May, and August of 2008, and were kept at − 80 °C until analysis (Granot et al. 2009). Central and specialized metabolites were measured by GC–MS and UPLC–QTOF-MS using a method for extraction modified from that previously described (Weckwerth et al. 2004). Briefly, 30 petioles from a mix of 50 random plants, distributed on the rocky slope, were homogenized using a pre-cooled mortar and pestle with liquid nitrogen; homogenized tissue, 100 mg, was milled for 2 min in a Mixer Mill 400 MM (25 frequency per sec, Retsch Gmbh & Co. KG, Germany) with two stainless steel grinding balls (5 mm). The ground tissue was extracted in a 1-mL pre-chilled methanol:chloroform:water solution (2.5:1:1 v/v) containing 380 µL of 1 mg/mL D-sorbitol-^13^C_6_ as an internal standard. The mixture was sonicated for 10 min and shaken for 10 min at 25 °C. After centrifugation at 20,817 ×*g* for 10 min, 300 µL of water and 300 µL of chloroform were added to the supernatant, vortexed and then centrifuged at 20,817 ×*g* for 5 min. The methanol–water phase was collected and transferred to another 2 mL tube and kept at − 80 °C until use. From each sample, 200 µL was dried in a vacuum concentrator (Eppendorf), derivatized, and subjected to the GC–MS analysis as previously described (Roessner et al. 2001; Hochberg et al. 2013).

### GC–MS data processing

The processing was done as previously described (Hochberg et al. 2013): Acquired spectra were searched against the National Institute of Standards and Technology (NIST, Gaithersburg, MD, USA) algorithm, incorporated in the Xcalibur^®^ data system (version 2.0.7), against RI libraries in the Max-Planck Institute of Plant Physiology in Golm, Germany (http://gmd.mpimp-golm.mpg.de/). The relative abundance of metabolites was calculated according to peak height and was normalized by the median and the sample fresh weight.

### UPLC–QTOF-MS analysis of specialized metabolites

From the extracted samples, after filtration (0.2µm PTFE filter, acrodisc, PALL) 2 µL was injected into the Xevo™ QTOF-MS coupled with the Waters Acquity UPLC System equipped with an ESI interface operating in negative and positive mode. The conditions were exactly as previously described (Hochberg et al. 2013). Chromatographic separation was carried out on an ACQUITY UPLC BEH C18 column (100 mm × 2.1 mm, 1.7 μm) with an ACQUITY UPLC BEH C18 VanGuard pre-column (130 Å, 2.1 mm × 5 mm, 1.7 µm). The column and autosampler were maintained at 40 °C and 10 °C, respectively. Lock mass accuracy was ensured using leucine enkephalin. The MS conditions were set as follows—capillary voltage: + 3.0 keV; sampling cone voltage: 27 V; extraction cone voltage: 4 V; source temperature: 120 °C; desolvation temperature: 300 °C; cone gas flow: 50 L/h; desolvation gas flow: 650 L/h; and collision energy: 6 eV. For MS/MS spectra, collision energies were set at 25–50 eV; scan range was set at 70–1000 m/z. The mobile phase comprised 95% water, 5% acetonitrile, 0.1% formic acid (phase A), and 0.1% formic acid in acetonitrile (phase B). The solvent gradient program was conditioned at 100–60% solvent A over the first 8 min, 60–0% solvent A over the next 1 min, and a return to the initial 100% A for 3.5 min, and conditioning at 100% A for 2.5 min, such that a single run comprised 15 min.

### UPLC–QTOF-MS data processing

The processing was done using Waters Corp software (Milford, MA, USA) Masslynx 4.1, Markerlynx XS, EZinfo, Elemental Composition, and MassFragment. Data were acquired using MassLynx 4.1. Processing and analysis (peak detection, data mining, alignment, and normalization) of the data were performed by MarkerLynx XS software. Multivariate statistical analysis (PCA and OPLS-DA) was performed on mass spectral datasets using EZinfo software in Pareto scaling. Markers identified by EZinfo software were transferred into Elemental Composition software to obtain potential molecular structures for the markers. The online databases Chemspider and MassBank were used for putative annotation of metabolites. Final confirmation of chosen formulae was carried out using MassFragment software.

### Statistical analysis

Following MS analysis, data of each run of GC–MS and UPLC–QTOF-MS were normalized separately: response values were calculated by dividing the peak height of each chemical feature by the FW of the respective sample and by the median of 100 identified markers in the same chromatogram. A second normalization, aimed at integrating the different years, was done by dividing the response value of a feature in each sample by its own median across all years. Hierarchical clustering (HCL) was performed using the software package MultiExperiment Viewer (MeV, v. 4.9.0) (Saeed et al. 2003), with the default weighted covariance-estimation function. The normalized data of all years were log-transformed for principal component analysis (PCA) using JMP software (v.12)**.** The correlation coefficients between metabolites and the environmental parameters, including precipitation, temperature, and relative humidity, were calculated by R (R v1.1.383 in RStudio), using the Pearson correlation in the corrplot package (McKenna et al. 2016).

## Results

### GC–MS-based metabolite profiling

**Fig. 2:**
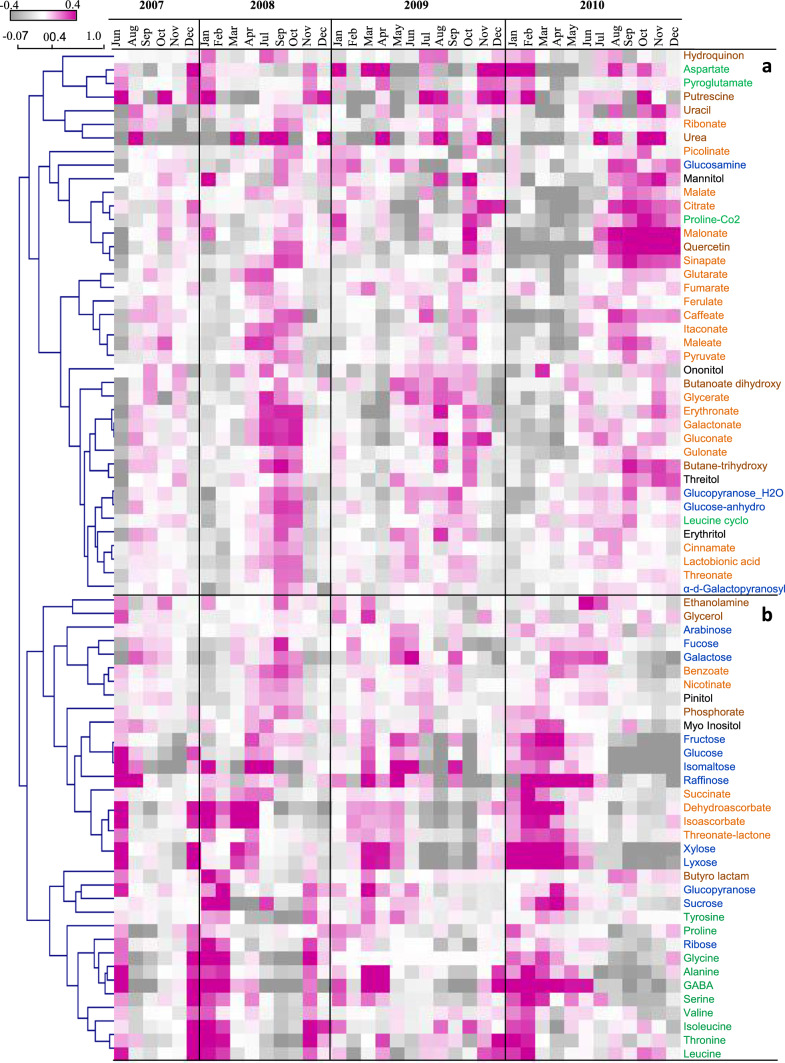
Hierarchal cluster analysis (HCL) of the relative content of the metabolites identified by GC–MS for 2007–2010. Different compound classes are presented on the heatmap in different colors: amino acids in green, sugars in blue, organic acids in orange, sugar alcohols in black, other in brown. (Missing months July 2007, April, May, and August 2008)

A set of 74 metabolites of the central metabolism was identified and confirmed across the study. The multiple-year results were integrated and analyzed for their pattern of change using hierarchal cluster analysis (HCL) (Fig. 2). The results show similar patterns of change in the relative abundances of metabolite groups, associated with specific periods of the year. These patterns are maintained despite (a) the yearly climate variability (Fig. 1b, c) and (b) the origin of the samples, collected from different randomly selected plants growing naturally on the rocky slopes.

In general, there are two leading patterns of change in the metabolite level across the year (Fig. 2). One is represented by Cluster a, which includes metabolites that are generally dominant in warmer and drier months; the second is represented by Cluster b, which includes metabolites that are abundant in cooler periods.

Specifically, Cluster a comprises organic acids, sugar derivatives, amino acid derivatives, and polyols. The organic acids include the TCA-cycle-related intermediates pyruvate, citrate, malate, and fumarate, and itaconate derived from aconitate and malonate derived from oxaloacetate. Succinate, another metabolite associated with the TCA cycle, appears in Cluster b, accumulating during the winter. In addition, precursors of the phenylpropanoid pathway, namely, caffeate, ferulate, cinnamate, and sinapate, were also placed in Cluster a. We also observed that erythronate, threonate, gluconate, gulonate, galactonate, and glycerate had a similar pattern, along with the polyols threitol, erythritol, onoitol, mannitol, and flavonol quercetin in Cluster a (Fig. 2).

In Cluster b, from winter to spring, there was an increase in the major sugars, fructose, glucose, and sucrose, and in several other sugars, raffinose, isomaltose, lyxose, xylose, ribose, and glucopyranose. Similar behavior was found with the sugar alcohol myo-inositol and three organic acids dehydroascorbate, isoascorbate, and succinate. That was not the case for the derivates of glucose, glucosamine, glucopyranose-H2O, and glucose anhydro, which only increased in the summer (in Cluster a).

In addition to the above, Cluster b was particularly enriched with amino acids, which accumulated at the end of autumn and during the winter, namely Ile, Leu, Val, Ser, Tyr, Thr, Pro, Ala, Gly, and GABA. There was a similar pattern in Cluster a for Asp and pyroglutamate. The results of Phe and Trp were similar to other amino acids, and were found in the GC–MS (data not shown) and, with higher resolution, in the UPLC–QTOF-MS. That was not the case for the amino acid derivatives, Pro-CO2 and Leu-cyclo, which increased in the summer (in Cluster a).

**Fig. 3:**
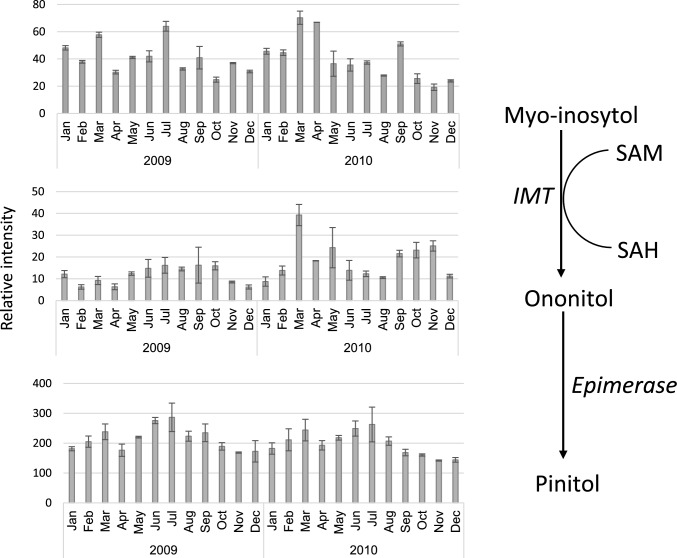
Level of pinitol, myo-inositol, and ononitol in 2009–2010 after normalization to median and fresh weight

Cluster b also includes the most dominant metabolite in *Z. dumosum*, namely, pinitol, belonging to the polyol group, which was consistently high, particularly from June to July in every year (Fig. 3, Supplementary Fig. s1). Some of the metabolites were higher from spring to summer in this cluster, such as arabinose, ethanolamine, glycerol, fucose, galactose, benzoate, and nicotinate, with a great deal of variability.

**Fig. 4:**
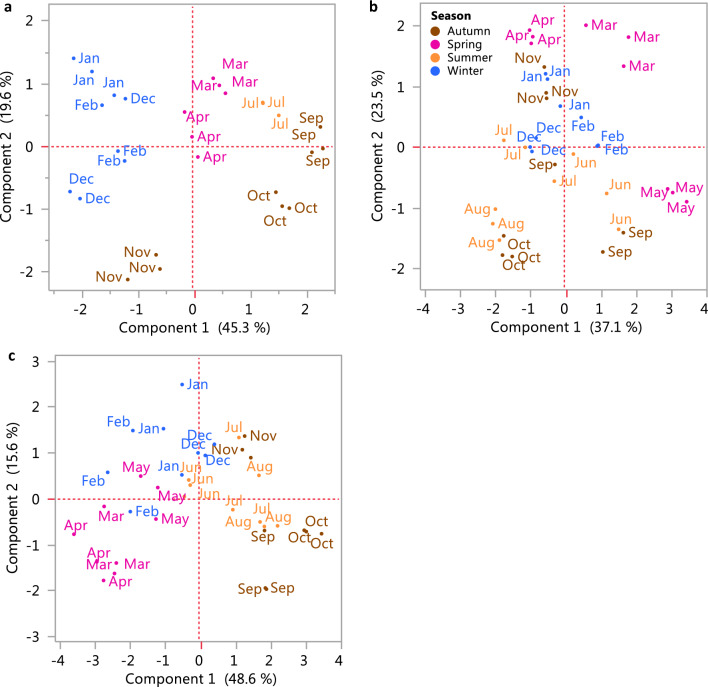
PCA of GC–MS results of three plant reproductive cycles, **a** from Nov 2007 to Oct 2008, **b** from Nov 2008 to Oct 2009, **c** from Nov 2009 to Oct 2010. Different seasons are presented on the PCA: winter in blue, spring in pink, summer in orange, autumn in brown

A principal component analysis (PCA) of the normalized metabolite data was performed to estimate the plant reproductive cycles (November to October) with regard to changes in petiole metabolism (Fig. 4a, c) (PCA of all metabolite data from 2007 to 2010, Supplementary Fig. s3). The variance of the metabolic matrix is reflected by the separation of colder and warmer months on PC1, with the exception of 2009, in which the colder months appear in the center of the plot and the warmer months are mostly separated on PC2. This exception is likely the result of the dataset variance in that year, being evenly explained by more than the two components presented.

The metabolite with the highest eigenvector in all plant reproductive cycles (November to October) is urea. Between the other metabolites with highest eigenvalues in the first reproductive cycle, were mostly amino acids GABA, Gly, Thr, Ile, Tyr, and Ala, and also dehydroascorbate, ribonate, and sucrose. The second and third cycles included mostly sugars, as well as related putrescine and GABA. All of these metabolites contribute to distinguishing between samples from the colder, wet months and those from warmer, dry months (Table 1).

**Table 1:** The 10 m/z metabolites with the highest eigenvectors in PC1 in the PCA of each of the three plant reproductive cycles from November to October (GC–MS data)

	Nov2007–Oct2008	PC1	Nov2008–Oct2009	PC1	Nov2009–Oct2010	PC1
1	Urea	0.48	Urea	− 0.54	Urea	0.53
2	GABA	− 0.45	Putrescine	− 0.41	Raffinose	− 0.31
3	Gly	− 0.26	Asp	− 0.41	Isomaltose	− 0.31
4	Thr	− 0.23	Isomaltose	0.31	Quercetin	0.29
5	Ile	− 0.18	Raffinose	0.18	GABA	− 0.21
6	Dehydroascorbate	− 0.18	Fructose	0.17	Glucose	− 0.19
7	Ribonate	0.14	Glucose	0.17	Malonate	0.19
8	Tyr	− 0.14	Lyxose	0.16	Xylose	− 0.17
9	Ala	− 0.13	Xylose	0.15	Lyxose	− 0.17
10	Sucrose	− 0.13	Dehydroascorbate	0.14	Citrate	0.16

**Fig. 5:**
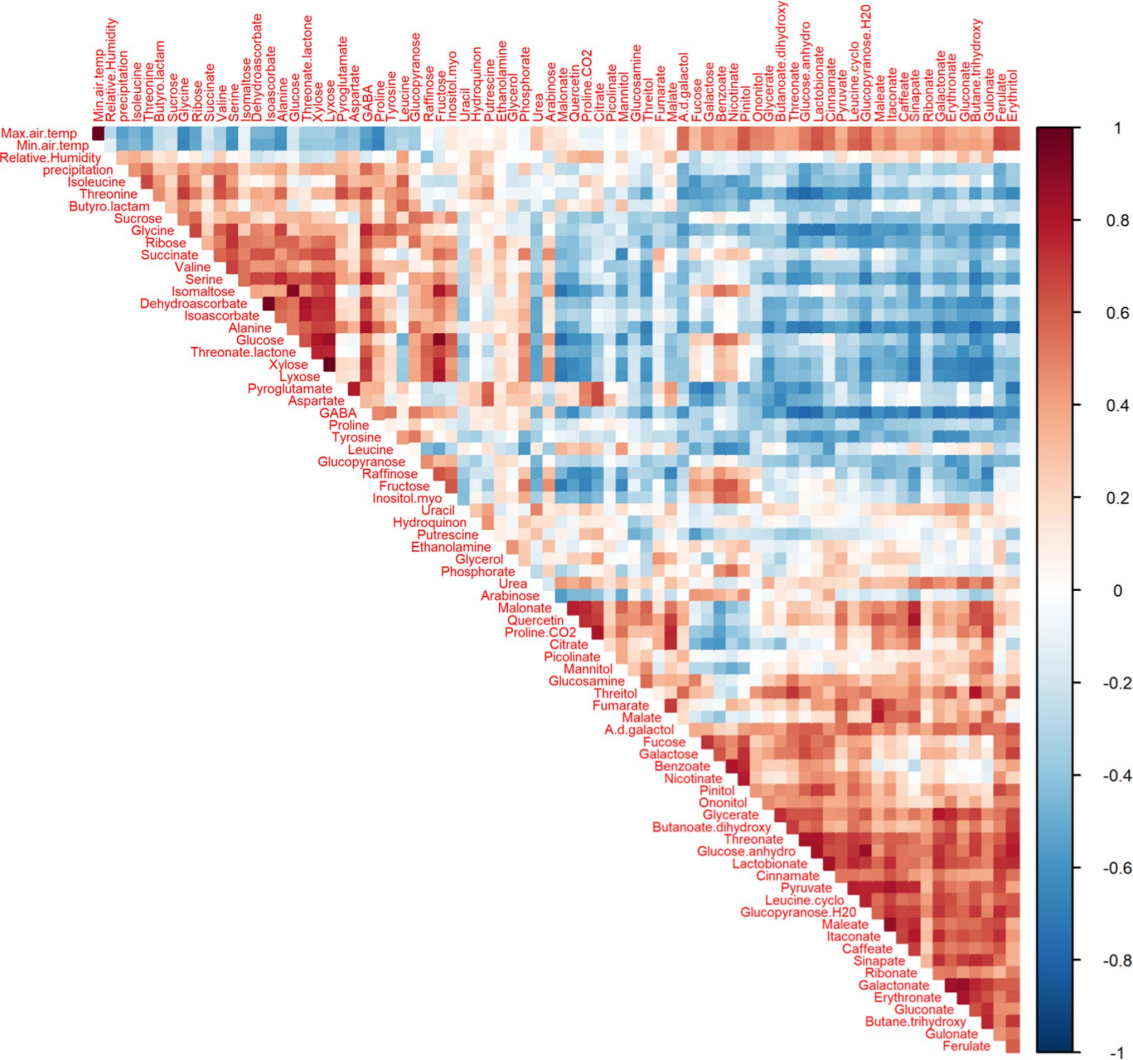
Correlation analysis of the relative contents of the primary metabolites identified by GC–MS from 2007 to 2010 with max and min temperature, relative humidity, and precipitation. Positive correlation in red, negative correlation in blue. The analysis was generated in R software

Supporting the HCL analysis, a correlation analysis (Fig. 5) showed the occurrence of two groups of metabolites, each with high positive “within” correlations and negative “between” correlations: group 1 includes amino acids and main sugars, and group 2 includes organic acids and polyols. Additionally, group 1 showed negative correlations with temperature, but positive ones with rainfall, while the inverse was shown for group 2. Both clusters had low correlations to relative humidity.

### UPLC–QTOF-MS analysis

**Fig. 6:**
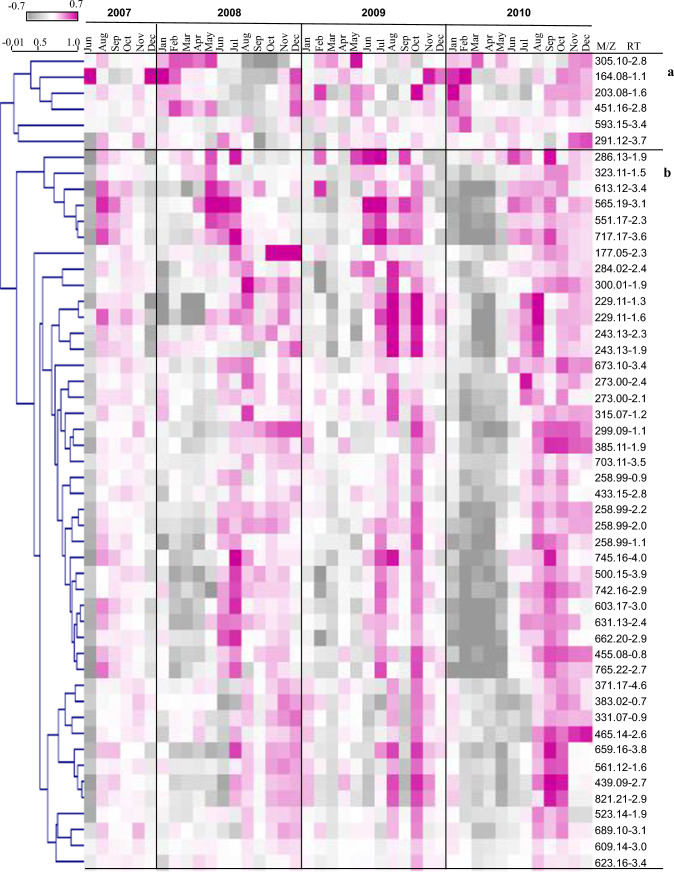
Hierarchal cluster analysis (HCL) of the relative contents of the m/z and tentative metabolites identified by UPLC–MS-QTOF for 2007–2010

In the negative and positive mode, 51 markers, some being putatively annotated metabolites (Supplementary Table. s1–s2), were consistently identified across the years. Normalized results of the mass-to-charge ratio (m/z), analyzed by HCl, showed two main patterns of change in metabolite abundance repeating across the years (Fig. 6). Mostly, specialized metabolites accumulated between summer and autumn (Cluster b). In Cluster a, two amino acids, Phe and Trp, increased mainly during the winter and decreased toward the summer in a pattern similar to the other amino acids identified by the GC–MS. Dominant metabolites in the UPLC–MS profiles were identified as sulfate compounds, flavonoid derivatives, and derivatives of piperazine.

**Fig. 7:**
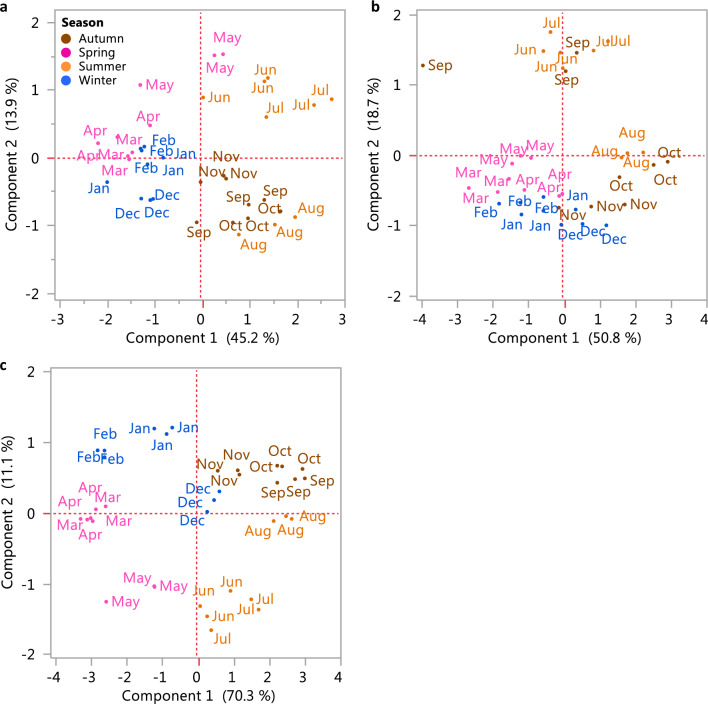
PCA of UPLC–QTOF-MS results from three plant reproductive cycles, **a** from Nov 2007 to Oct 2008, **b** from Nov 2008 to Oct 2009, **c** from Nov 2009 to Oct 2010. Different seasons are presented on the PCA: winter in blue, spring in pink, summer in orange, autumn in brown

A PCA of the normalized m/z data from the UPLC–QTOF-MS results was performed to estimate plant reproductive cycles (November to October) with regard to changes in petiole metabolism (Fig. 7a–c) (PCA of all m/z data from 2007 to 2010, Supplementary Fig. s4). The variance of the metabolic matrix was reflected mainly by the seasons (Table 2). The m/z 229.12, putatively 1,4-piperazinedipropanoate, had highest eigenvector in the first and second reproductive cycle.

**Table 2:** The 10 m/z markers with the highest eigenvectors in PC1 in the PCA of each of the three plant reproductive cycles from November to October (UPLC–QTOF-MS data, m/z and retention time)

	Nov2007–Oct2008	PC1	Nov2008–Oct2009	PC1	Nov2009–Oct2010	PC1
1	229.12–1.6	0.32	229.12–1.3	0.26	662.21–2.9	0.23
2	229.12–1.3	0.32	229.12–1.6	0.25	765.23–2.7	0.23
3	500.16–3.9	0.24	243.13–2.3	0.25	455.09–0.8	0.21
4	164.07–1.1	− 0.21	243.13–1.9	0.23	631.13–2.4	0.21
5	745.16–4.0	0.21	439.09–2.7	0.20	603.17–3.0	0.21
6	631.13–2.4	0.21	659.16–3.8	0.20	243.13–2.3	0.21
7	603.17–3.0	0.21	821.22–2.9	0.19	258.99–2.0	0.20
8	717.17–3.6	0.20	299.09–1.1	0.18	717.17–3.6	0.20
9	305.11–2.8	− 0.20	745.16–4.0	0.18	613.12–3.4	0.20
10	258.99–2.0	0.19	300.02–1.2	0.18	742.17–2.9	0.19

**Fig. 8:**
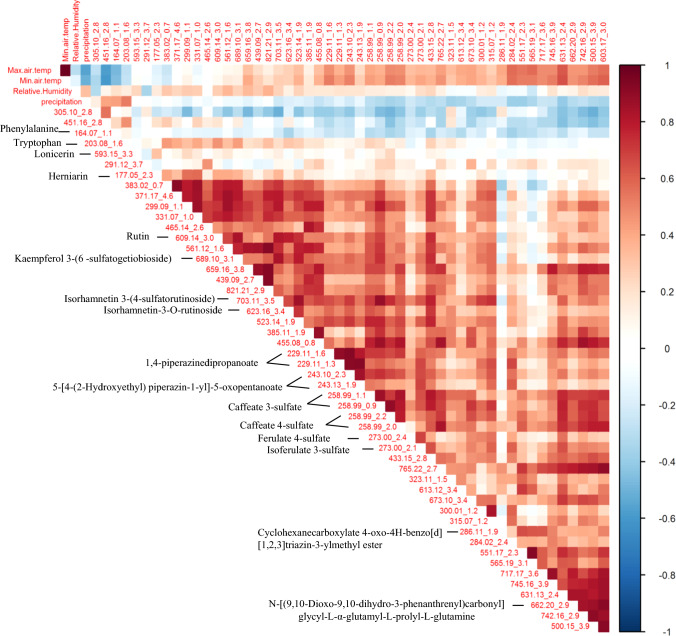
Correlation analysis of relative contents of the secondary metabolites (m/z and retention time) and the putatively annotated m/z from the UPLC–QTOF-MS data. The correlation of years 2007–2010 with max and min temperature, relative humidity, and precipitation. Positive correlation in red, negative correlation in blue. The analysis was generated in R software

A correlation analysis between the m/z markers and climate (Fig. 8) parameters showed a high positive correlation between most of the m/z markers and the average daily temperature of each month. The correlation with precipitation was low and negative, while the correlation with relative humidity was low but positive. A different pattern of correlation was shown for Phe, Trp, and unknown marker 451.16, having a high positive correlation with precipitation, a low correlation with relative humidity, and a negative correlation with temperature (as found for other amino acids measured by GC–MS).

**Fig. 9:**
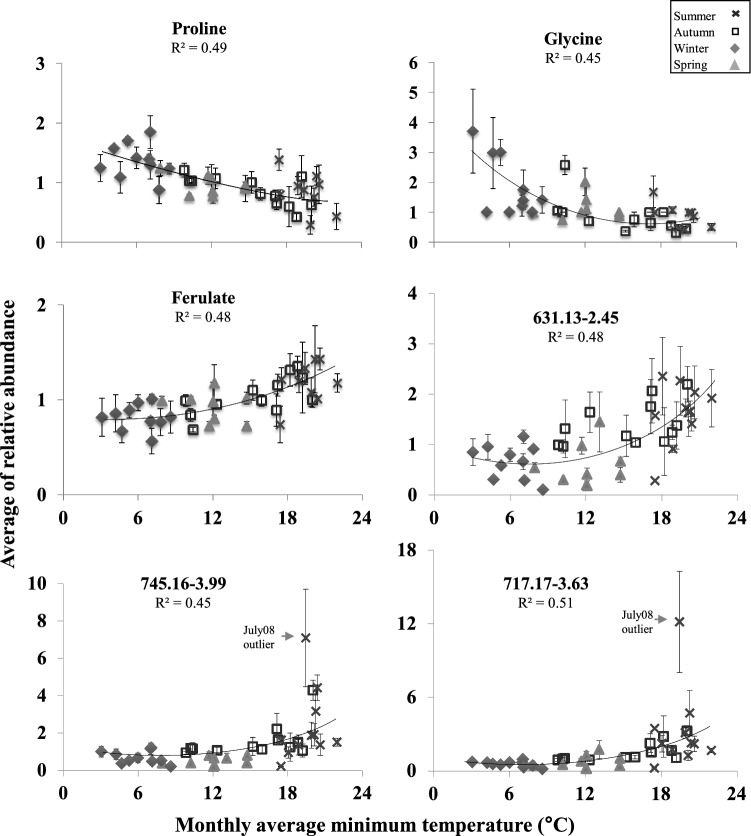
Regression analysis of average monthly content of central and speclialized  metabolites to minimum temperature (0.5 > *R*^2^ > 0.4). Different seasons are presented by winter in diamond, spring in triangle, summer in x, autumn in square

The regression model (Fig. 9) showed only a low relation between metabolites and different environmental parameters, *r*^2^ < 0.5. Notably, only temperature could explain metabolites pattern of change with regression coefficient higher then *r*^2^ > 0.45, (*p* > 0.005) (Fig 9 presents the regression against minimum temperature only, as the model for maximum temperature was very similar). Among the metabolite profiles explained by the regression model, the amino acids Pro and Gly displayed the highest relative abundances in the colder months (*r*^2^ > 0.45) when the temperatures dropped below 8 °C  at night. In contrast ferulate and m/z 717.17, 631.13  and 603.17 accumulated during the summer and autumn when minimum  temperatures were higher then 16 °C.

## Discussion

*Zygophyllum dumosum Boiss* is a dominant plant on the southeast-facing slopes of the Negev Desert in Israel. The natural environment imposes a combination of abiotic stresses on this plant species, including drought, heat, and salinity, particularly during the hot season. One way in which the plant avoids drought is through its semi-deciduous phenology in which leaflets senesce and die at the beginning of the summer, while leaving the petioles alive but dormant and essentially photosynthetically inactive during the dry season (Terwilliger 1994).

Our study characterizes the monthly pattern of change in the metabolism of *Z. dumosum* petioles over a period of 3.5 years. For this purpose, we applied mass-spectrometry-based metabolite analysis and followed the changes in metabolite profiles, testing the level of coordination in the central and specialized metabolism, in response to contrasting seasonal climates, and the yearly variance.

Only a few studies have compared seasonal and yearly changes in leaf metabolism. In one of these studies, in the Patagonian shrub *Larrea divaricata*, the total phenols were analyzed in leaves and were shown to be relatively high during the dry summer and autumn, and lower during the wet winter and spring. Moreover, a significant relationship was shown between total phenols and antioxidant capacity (Varela et al. 2016, 2018). In a different study that was carried out on the plant *Tithonia diversifolia*, it was found that each of the plant parts, namely, the inflorescence, root, leaf, and stem, responded differently to changes in environmental conditions. Comparing the organs across 2 years in two different regions, the authors established that the inflorescence and root metabolites were mainly affected by variation in soil nutrients, while the leaf and stem were mainly affected by seasonal variation, primarily by rainfall and humidity. Their results showed that during the rainiest periods, central metabolites, such as sugars and nucleosides, were enriched; in contrast, the drier periods, particularly during the cold months, were rich in specialized metabolites (Sampaio et al. 2016). Our multi-year study of *Z. dumosum* petioles shows the occurrence of a seasonal pattern in metabolites that, generally speaking, repeated across the years. The correlation analysis highlighted the relation between metabolites and temperature and precipitation but showed only minor correlations with relative humidity or radiation and wind speed (correlation data for  radiation and wind speed not shown). Among the central metabolites, we observed amino acids increasing at the beginning of the winter; the major sugars accumulated during winter and spring and were metabolized during hot and dry periods; notably, the known stress metabolites GABA, raffinose, and ascorbate (Xu et al. 2021; Yan et al. 2022; Jia et al. 2020) were found in abundance in months characterized by a milder climate. In contrast, organic acids, including TCA cycle intermediates, polyols, phenylpropanoids, sulfate conjugates, and piperazines accumulated in the warmer, drier season.

### Is the shift in metabolite composition associated with environmental cues or is it a developmental process?

The accumulation of groups of metabolites in the dry months may be due to a response to environmental cues such as drought and radiation. Water deficit is known to lead to the accumulation of compounds in plant organs. For example, carbohydrates can accumulate in the forms of minor sugars, organic acids, or phenolic compounds (Bresta et al. 2018). As a response to stress, metabolites may act in different ways, as a pool of energy resources or as mediating osmotic adjustment, antioxidants, and signaling (Bresta et al. 2018; Igamberdiev and Eprintsev 2016; Moing 2000; Lopez-Bucio et al. 2000; Kaur and Meena 2019). Moreover, the biosynthesis of many specialized metabolites is induced by radiation in the skin of fruits such as tomato and grape berries (Reshef et al. 2017; Abreu et al. 2019; Romero-Roman et al. 2021) and in the leaf tissues in *Secale cereale*, olive, and bell pepper (Reuber et al. 1996; Lorini et al. 2021; Ellenberger et al. 2020). Radiation and temperature, which increase in the desert during the summer months, can lead to a severe increase in plants’ surface temperature. In grape berry skin exposed to solar radiation, the temperature reached 45–50 °C on a daily basis. Such temperatures can reduce the activity of photosynthetic tissues, and can modulate polyphenol metabolism and the accumulation of organic acids and sugars (Reshef et al. 2017; Reuber et al. 1996). In our study, specialized metabolites increased during the summer, including strong antioxidants, such as pinitol, caffeic, ferulic acids, and quercetin, with a known role in adaption to abiotic stresses (Ramakrishna and Ravishankar 2011; Fabregas and Fernie 2019; Gharibi et al. 2019). Moreover, an increased content of newly identified sulfated metabolites (Sikron-Persi et al. 2019) was also recorded. Sulfated metabolites were shown to be involved in stomatal aperture control in Arabidopsis (Teles et al. 2018); however, more common sulfated metabolites, such as glutathione, methionine, and cysteine, were not detected.

The increase of metabolites of the phenylpropanoid pathway, observed during the summer, may be the result of cell wall lignification, a plant defense mechanism against abiotic stresses (Cesarino 2019). For instance, we showed that hydroxycinnamic acids increased in lignified petioles collected in the dry season, while they decreased in the green non-lignified petioles in winter. Consistent with our findings, Schulze showed that the dormant-like stage, including the lignification of petioles, can be delayed by improving the plant’s water status (Schulze et al. 1980; Waisel et al. 1970). Taken together, these results imply that water availability is a major factor governing petiole phenology and metabolism in *Zygophyllum*.

Dormancy can also induce metabolite shifts. During bud dormancy in sweet cherry trees, phenolics accumulate until the release of dormancy; in a similar manner, the sugars fructose and glucose are low upon leaf shedding at the beginning of endodormancy and increase until dormancy is released (Baldermann et al. 2018). The relevance of metabolite accumulation, due to the physiological stage of dormancy, can be shown by the consistent changes in metabolic patterns irrespective of the severity of a specific year’s environmental conditions. Dormant seeds and buds, in summer or winter, share many characteristics (Gillespie and Volaire 2017); for example, in apple buds, it was shown that nitrogen compounds, including amino acids, increased during dormancy, reaching maximum levels just prior to bud growth (Seif El-Yazal et al. 2013). Similarly, in our study, the amino acids increased during the shift from the dry to the wet season; at this time, petioles increase in size and change from grey to green as a result of chlorophyll accumulation and photosynthetic activity. Additionally, metabolic changes can be related to organ function and source to sink relations, such as mature leaves, which are a source of carbohydrates and nitrogen for roots, flowers, fruits, and young shoots (Baslam et al. 2020; Fan et al. 2019; Zhang et al. 2021; Ruan and Gerendás 2015).

In citrus in the spring, 70% of nitrogen that transport from old reserve organs is repartitioned to the new developing organ (Legaz et al. 1995). Source–sink relations might also explain the metabolic changes in *Zygophyllum* petioles; during the winter, (i) old petioles function as source tissues for new shoot growth, in leaves and flowers, thus decreasing the free metabolite pool, and (ii) carbon moieties are replenished as a result of photosynthesis (Igamberdiev and Eprintsev 2016).

Lastly, polyols, among them the dominant pinitol, are found at relatively high levels throughout the year and further increase during the summer, and they may function as a defense mechanism against drought. Early work described a desiccation/stress-related cDNA isolated from the halophyte *Mesembryanthemum crystallinum* encoding for a myo-inositol O-methyltransferase (*Imt1*), which catalyzes the first step in the biosynthesis of the cyclic sugar alcohol, pinitol (Vernon and Bohnert 1992). IMT1 overexpression in *Nicotiana tabacum* or *Arabidopsis thaliana* resulted in the accumulation of D-ononitol, the precursor of D-pinitol, and increased drought and salt tolerance (Sheveleva et al. 1997; Ahn et al. 2018). Furthermore, pinitol biosynthesis was enhanced in the leaves of drought-tolerant genotypes of soybean under control conditions compared to sensitive genotypes (Silvente et al. 2012; Ramakrishna and Ravishankar 2011; Fabregas and Fernie 2019; Gharibi et al. 2019; Sayed 1996). A role for pinitol in osmotic adjustment was suggested in the Leguminosae family, where it was found to increase in response to abiotic stress.

## In summary

From the multiple-year time series analysis of the metabolite profiles of *Z*. *dumosum* petioles, we have shown a functional partitioning in metabolic composition between seasons. The question remains as to whether these processes are induced by the environment or are part of a conserved shift tightly bound to plant phenology, as a perennial adaptation to seasonal changes. We tend to conclude that it is combination of the two. The phenology-induced role seems to be a dominant factor, as suggested by the consistent changes during successive years characterized by different environmental conditions. Future studies should explore the regulation of *Zygophyllum* metabolism at the transcriptional level, to resolve the basis of the seasonal rhythms identified. In this article, we focused on petioles only; in the future, we would like to expand the understanding of the mechanism in which the plant works under the various conditions. We will follow the synthesis of the metabolites in the different parts of the plant up to the petiole using isotope-labeled substances and we will test the effect of different stresses on the growth of the plants under controlled conditions. Furthermore, efforts should be put to study the whole-plant seasonal acclimation, its physiology, and metabolism beyond the single organ, the petiole.

### *Author contribution statement*

GGrafi, NSP, and AF designed the experiment; GGrafi and AF supervised the research. GGrafi, GGranot, and NSP collected the samples; GGrafi provided the digital images of the plant; NSP was responsible for the metabolite profiling; AB and DT worked on the statistical analysis of the metabolic data with NSP. AF and NSP interpreted the results and wrote the manuscript. All authors read, edited, and approved the manuscript.

## Supplementary Information

Below is the link to the electronic supplementary material.

Supplementary file1 (DOCX 297 KB)

## Data Availability

The datasets generated during and/or analyzed during the current study are available from the corresponding author on reasonable request.

## Abbreviations

GC–MS: Gas chromatography–mass spectrometry
UPLC–QTOF-MS: Ultra-high performance liquid chromatography–quadrupole time-of-flight mass spectrometry
