# Supplementary material for: Mass spectrometry-based metabolite profiling reveals functional seasonal shifts in the metabolome of Zygophyllum dumosum Boiss and its relation to environmental conditions
Source: Planta. 2023 Jun 3;258(1):10. doi: 10.1007/s00425-023-04168-2 (PMC10239397; doi:10.1007/s00425-023-04168-2)
Supplement: Supplementary file 1 — Supplementary file1 (DOCX 297 KB) [file 425_2023_4168_MOESM1_ESM.docx]

**Supplementary Material**

**Mass spectrometry-based metabolite profiling reveals functional seasonal shifts in the metabolome of *Zygophyllum dumosum* *Boiss* and its relation to environmental conditions**

[**Noga Sikron-Persi**](http://www.springerlink.com/content/?Author=Noga+Sikron-Persi)**^1^, Gila Granot^1^, Albert Batushansky^1,2^, David Toubiana^1^, Gideon Grafi^1^, and Aaron Fait Mails^1,#^**

^1^ French Associates Institute for Agriculture and Biotechnology of Drylands, Jacob Blaustein Institutes for Desert Research, Ben-Gurion University of the Negev, Sde Boker Campus, 84990, Midreshet Ben-Gurion, Israel.

^2^ Ilse Katz Institute for Nanoscale Science & Technology, Ben-Gurion University of the Negev, Beer Sheva, 84105, Israel

^*^ Corresponding to:

Aaron Fait mails, [fait@bgu.ac.il](mailto:fait@bgu.ac.il) ; Telephone 972-502029629; Fax 972-86596742


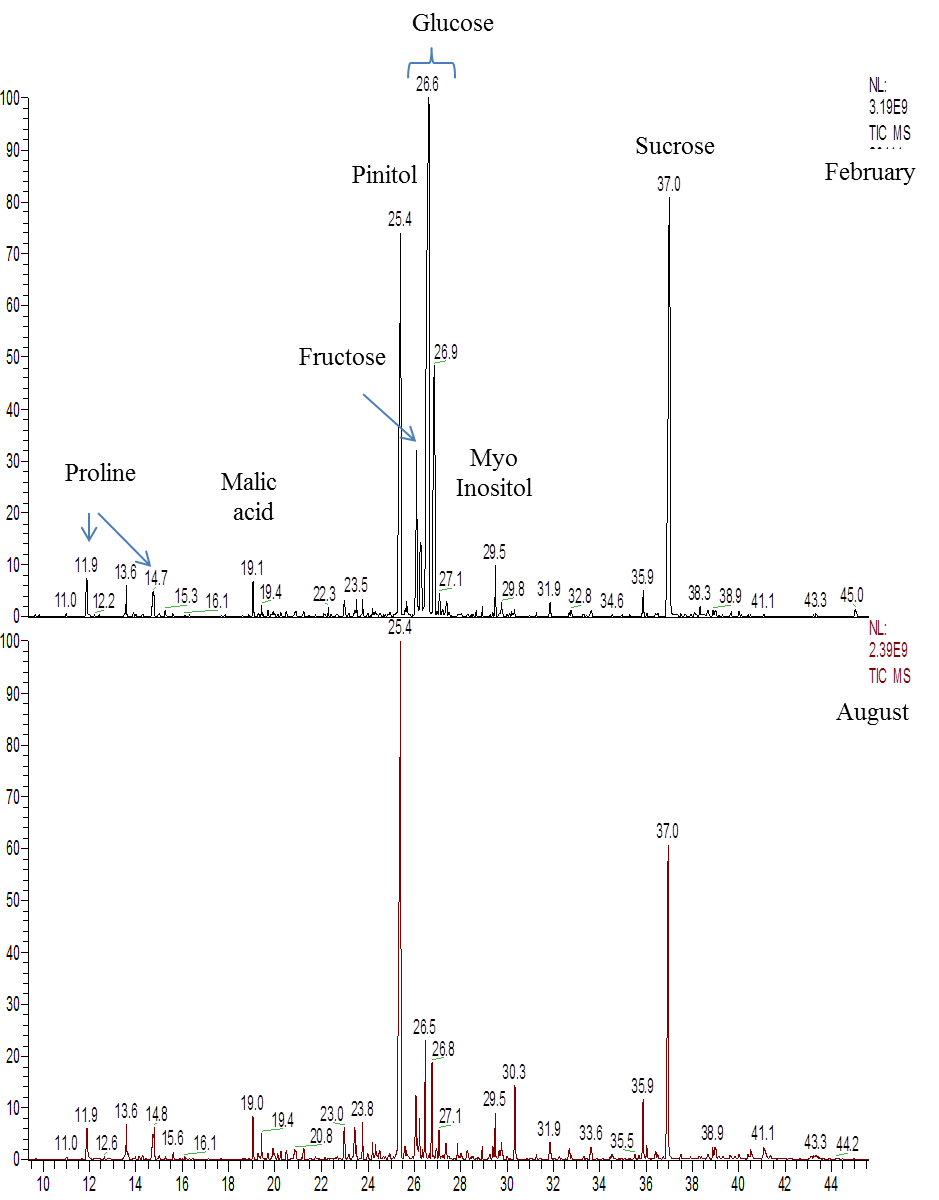


**b**

**a**

**Fig. s1**  GC-MS chromatogram showing changes in pinitol between winter and summer. **a** February, representative of winter and **b** August, representative of summer.

Relative humidity (%)

**b**

**Fig. s2**. Average of monthly relative humidity (%) during 2006–2010 at Sde Boker.

**a**

**b**

**Fig. s3**. PCA of GC-MS results. **a** analysis of monthly primary metabolites (Jun 2007 to Dec 2010) Different seasons are presented on the PCA: winter in blue, spring in pink, summer in orange, autumn in brown. **b** loadings plot.

**a**

**b**

**Fig. s4**. PCA of UPLC-QTOF-MS results: **a**: analysis of monthly primary metabolites (Jun 2007 to Dec 2010). Different seasons are presented on the PCA: winter in blue, spring in pink, summer in orange, autumn in brown. **b** loadings plot.

**Table. s1 :**Dominant metabolites tentatively annotated from the UPLC-Q-TOF-MS data.

| **Rt(min)** | **MW** | **M+H** | **Fragment+** | **M-H** | **Fragment-** | **Formula** | **Name** |
| --- | --- | --- | --- | --- | --- | --- | --- |
| 0.88+1.01  2.07+2.18 | 259.99 | **261.00** | 242.99 163.03 | **258.99** | 179.03 134.04 | C_9_H_8_O_7_S | Caffeate 4-sulfate  Caffeate 3-sulfate |
| 131+.1.61 | 230.261 | **231.13** | 139.12 185.12 | **229.13** | 114.05 | C_10_H_18_N_2_O_4_ | 1,4-piperazinedipropanoate |
| 1.64+1.88 | 287.314 | **288.13** | 177.05 112.08 | **286.11** |  | C_15_H_17_N_3_O_3_ | Cyclohexanecarboxylate 4-oxo-4H-benzo[d][1,2,3]triazin-3-ylmethyl ester |
| 1.94+2.3 | 244.287 | **245.15** | 199.14 153.13 | **243.13** | 114.05 | C_11_H_19_N_2_O_4_ | 5-[4-(2-Hydroxyethyl) piperazin-1-yl]-5-oxopentanoate |
| 2.11  2.40 | 274.247 |  |  | **273.00** | 193.05 178.02 134.03 | C_10_H_10_O_7_S | Ferulate 4-sulfate  Isoferulate 3-sulfate |
| 2.2+2.37 | 176.169 | **177.05** | 145.02 117.03 |  |  | C_10_H_8_O_3_ | Herniarin |
| 2.88 | 663.63 | **664.22** | 355.117 337.10 | **662.20** | 353.1 | C_32_H_33_N_5_O_11_ | N-[(9,10-Dioxo-9,10-dihydro-3-phenanthrenyl)carbonyl]glycyl-L-α-glutamyl-L-prolyl-L-glutamine |
| 3 | 610.517 | **611.16** | 465.10 303.05 | **609.14** | 301.03 300.02 | C_27_H_30_O_16_ | Rutin* |
| 3.1 | 690.581 | **691.22** | 545.058 | **689.09** | 387.06 | C_27_H_30_O_19_S | 5,7-Dihydroxy-2-(4-hydroxyphenyl)-4-oxo-4H-chromen-3-yl 6-O-(6-O-sulfo-β-D-glycero-hexopyranosyl)-β-D-glycero-hexopyranoside |
| 3.44 | 624.5441 | **625.17** | 479.11 317.06 | **623.16** |  | C_28_H_32_O_16_ | Isorhamnetin-3-O-rutinoside |
| 3.54 | 704.126 | **705.13** | 625.17 559.06 317.06 | **703.11** | 387.05 | C_28_H_32_O_19_S | Isorhamnetin 3-(4''-sulfatorutinoside) |

**Table. s1**. The dominant metabolites tentatively annotated from the UPLC-Q-TOF-MS data.

**Table. s2: Smiles of the metabolites tentatively identified.**

**Caffeic acid 3-sulfate**

c1cc(c(cc1/C=C/C(=O)O)OS(=O)(=O)O)O

**Caffeic acid 4-sulfate**

c1cc(c(cc1/C=C/C(=O)O)O)OS(=O)(=O)O

**1,4-piperazinedipropionate**

C1CN(CCN1CCC(=O)O)CCC(=O)O

**Cyclohexanecarboxylate 4-oxo-4H-benzo[d][1,2,3]triazin-3-ylmethyl ester**

c1ccc2c(c1)c(=O)n(nn2)COC(=O)C3CCCCC3

**5-[4-(2-Hydroxyethyl) piperazin-1-yl]-5-oxopentanoate**

C1CN(CC[NH+]1CCO)C(=O)CCCC(=O)[O-]

**Ferulic acid 4-sulfate**

COC1=C(C=CC(=C1)C=CC(=O)O)OS(=O)(=O)O

**Isoferulic acid 3-sulfate**

COC1=C(C=C(C=C1)C=CC(=O)O)OS(=O)(=O)O

**Herniarin**

COc1ccc2ccc(=O)oc2c1

**N-[(9,10-Dioxo-9,10-dihydro-3-phenanthrenyl)carbonyl]glycyl-L-α-glutamyl-L-prolyl-L-glutamine**

O=C(N[C@H](C(=O)O)CCC(=O)N)[C@H]4N(C(=O)[C@@H](NC(=O)CNC(=O)c3ccc2C(=O)C(=O)c1ccccc1c2c3)CCC(=O)O)CCC4

**Lonicerin**

CC1C(C(C(C(O1)OC2C(C(C(OC2OC3=CC(=C4C(=C3)OC(=CC4=O)C5=CC(=C(C=C5)O)O)O)CO)O)O)O)O)O

**Kaempferol 3-(6 sulfatogetiobioside)**

c1cc(ccc1c2c(c(=O)c3c(cc(cc3o2)O)O)O[C@H]4C(C([C@@H](C(O4)CO[C@H]5C(C([C@@H](C(O5)COS(=O)(=O)O)O)O)O)O)O)O)O

**isorhamnetin 3-O-robinobioside**

C[C@H]1[C@@H]([C@H]([C@H]([C@@H](O1)OC[C@@H]2[C@@H]([C@@H]([C@H](C(O2)Oc3c(=O)c4c(cc(cc4oc3c5ccc(c(c5)OC)O)O)O)O)O)O)O)O)O

**Isorhamnetin 3-(4-sulfatorutinoside)**

CC1C(C(C(C(O1)OCC2C(C(C(C(O2)OC3=C(OC4=CC(=CC(=C4C3=O)O)O)C5=CC(=C(C=C5)O)OC)O)O)OS(=O)(=O)O)O)O)O

**Table. s2**. SMILES of the dominant metabolites that were tentatively identified from the UPLC-QTOF-MS results.
